# Wound healing effect of *Euphorbia hirta linn.* (Euphorbiaceae) in alloxan induced diabetic rats

**DOI:** 10.1186/s12906-017-1930-x

**Published:** 2017-08-24

**Authors:** Riazul Haque Tuhin, Marium Begum, Sohanur Rahman, Rubaba Karim, Taslima Begum, Siraj Uddin Ahmed, Ronia Mostofa, Amir Hossain, Mohamed Abdel-Daim, Rayhana Begum

**Affiliations:** 1 Department of Pharmacy, Primeasia University, Dhaka, 1213 Bangladesh; 2 Department of Pharmacy, East West University, Aftabnagar, Dhaka, 1212 Bangladesh; 3 Graduate School of Innovative life Science, University of Toyama, 3190 Gofuku, Toyama, 930-8555 Japan; 4 Pharmacology Department, Faculty of Veterinary Medicine, Suez Canal University, Ismailia, 41522 Egypt

**Keywords:** *Euphorbia hirta*, Wound healing, Alloxan, Nitric oxide, Malondialdehyde

## Abstract

**Background:**

*Euphorbia hirta linn.*, is a species of Euphorbiaceae family. They are known as asthma plant, barokhervi. The plant *E. hirta* is famous for its medicinal importance among the tribal population. It is a common practice to use the whole to heal wounds. Several pharmacological properties including antiseptic, anti-inflammatory, antidibetic, antispasmodic, antibacterial, antiviral, antifungal, anticonvulsant, nootropic, antifertility and aphrodisiac properties have already been reported for this plant. The aim of present work was to evaluate the wound healing property in diabetic animals by oral and topical administration of ethanolic extract of *E. hirta* whole plant.

**Methods:**

The ethanolic extract of *E. hirta* was subjected to determine the total phenolic content and total flavonoid content using galic acid and quercetin, respectively as standard. A single injection of alloxan monohydrate (120 mg/kg, i.p.) prepared in normal saline was administered to produce diabetes in rats, after overnight fasting. For analyzing the rate of contraction of wound, excision wounds sized 4.90cm^2^ and of 2 mm depth were used. Oral (100, 200 and 400 mg/kg/day; p.o.) and topical treatment with the extract (5% and 10% ointment 50 mg/kg/day) and standard (5% povidone iodine ointment 50 mg/kg/day) was started on the day of induction of wound and continued up to 16 days. The means of wound area measurement between groups at different time intervals were compared using ANOVA and Dunnet’s test. The diabetic wound healing mechanism was studied by measuring the plasma level of glucose, malondialdehyde (MDA) and nitric oxide (NO) in both control and treated groups. For the confirmation of activity, histopathology of the wounds tissues from excision wound model was performed.

**Results:**

Phytochemical investigations showed the presence of various phytoconstituents (carbohydrates, saponins, alkaloids, glycosides, steroids, flavonoids, tannins). In the ethanolic extract of *E. hirta* the total phenol content was 285 ± 3.22 mg/g whereas the total flavonoid content was 118.46 ± 1.85 mg/g. In the present study, *E. hirta* caused significant wound closer both orally (35.92%, 44.69% and 61.42% at the doses of 100, 200 and 400, respectively) and topically (32.86% and 36.32% at the doses of 5% and 10%) treated groups as compared to diabetic control. However, the orally treated groups showed more significant effect than the topically treated groups. Moreover, oral administration of *E*. hirta ethanolic extract significantly reduced the blood glucose levels in diabetic wound rats (*p* < 0.01) on day 8 and day 16 as compared to the diabetic wound control (*p* < 0.01). On the other hand, topical application of *E. hirta* did not influence the blood glucose levels in diabetic rats (*p* > 0.05). It also demonstrated a significant decrease in the plasma levels of lipid malondialdehyde and nitric oxide. The results of biochemical parameters were further supported by the histopathological changes of different organs (liver, pancrease, kidney, heart and skin from wound area) which were evidenced through a decrease in inflammatory cell infiltration.

**Conclusion:**

The present study demonstrates that *E. hirta* whole plant extract promotes healing of wounds more significantly as compared to diabetic control rats, where healing is otherwise delayed.

## Background

Wound may be defined as a loss or breaking of cellular and anatomic or functional continuity of living tissue [1] which is generally classified according to the depth of tissue loss. Wounds are recognized as one of the major problems in developing countries resulting severe complications in many cases that leads to high cost for therapy [2]. The healing process generally occurs in three stages including inflammation, proliferation, and remodeling depending upon the extent of damage and the host’s ability to repair tissue [3]. Improper wound healing control may give rise to diabetic foot ulcer or amputation in extreme cases [4]. According to the WHO report, diabetes is set to elevate from 171 million to 366 million worldwide by the year 2030 [5]. It is believed that the main reason for the increased rate in morbidity and mortality of diabetes is due to the development of macro- and micro-vascular complications which include failure of the wound healing process [6]. Poorly repaired wound may give rise to sequence of events involving inflammation, proliferation and migration of different cell types [7]. However, recovery of the early phase of wound (inflammatory phase) will greatly influence the overall integrity of the healing process [8]. Former studies showed that high glucose concentration leads to formation of sugar-derived substances called advanced glycation end products (AGEs) that inhibit the wound healing process by delaying inflammatory and proliferation phases of the process [6]. This eventually leads to generation of free radicals resulting imbalance in the level of free radicals and antioxidants which induces oxidative stress and tissue damage followed by delayed wound healing [9]. Therefore, elimination of ROS (reactive oxygen species) could be a significant strategy in healing wounds [10].

*Euphorbia hirta linn.* (Family: Euphorbiaceae, Species: *Euphorbia hirta*) is a small, erect or ascending annual herb reaching up to 50 cm, with hairy stems. It is known as baridudhi in Hindi and barokhervi in Bengali [11]. *Euphorbia hirta linn.* is an official drug included in African pharmacopeia of 1985 [12]. *E. hirta* is distributed throughout the hotter parts of India and Australia where it is often found in waste places along the roadsides [13]. In traditional Indian medicinal systems, leaves of *Euphorbia hirta* used in the treatment of coryza, cough, asthma, bronchial infections, bowel complaints, helminthic infestations, wounds, kidney stones and abscesses [11]. The aqueous extract exhibits anxiolytic, analgesic, antipyretic, and anti-inflammatory activities [11]. Preliminary phytochemical screening of ethanolic extract of *Euphorbia hirta* shows the presence of plant tannins, flavonoids, alkaloids, cardiac glycosides along with other anti-oxidants and absence of saponin, cyanogenic glycosides [14].

In traditional medicine, herbal-based drugs have been reported to exhibit curative value for various disorders [15]. For many years, plants have been used as the remedy for various skin and dermatological disorders especially cut, wounds, burns etc. [16]. Although distinct treatment options are available (analgesics, antibiotics, anti-inflammatory drugs etc) for wound healing management, most of these therapies are responsible for undesirable side effects [17]. Herbal medicines play promising role in wound management by facilitating disinfection, debridement and provision of suitable environment for the natural healing process [18]. These medications are less toxic and provide fewer side effects compared to that of conventional medications. Therefore, it is important to establish a scientific validation for the medicinal effect of plants as traditional medicine in wound healing [2].

The evidence of antidibetic properties of *E. hirta* have already been reported in the previous study [19]. Moreover, due to the presence of flavonoid compounds in the whole plant extract it was found to induce anti-inflammatory and wound healing effects [20, 21] which lead us to assess the diabetic wound healing activity of *E. hirta*. Hence, the present work involves evaluation of wound healing property in alloxan induced diabetic animals by oral and topical administration of ethanolic extract of *E. hirta* whole plant.

With regards to the above mentioned facts, the present study was therefore, undertaken to evaluate the wound healing property in alloxan induced diabetic animals by oral and topical administration of ethanolic extract of *E. hirta* whole plant.

## Methods

### Plant material

Whole plants of *Euphorbia hirta* were collected on May, 2015 from the road side (Road 83, Gulshan 2, Dhaka) situated in front of Saudi Arabian Embassy in Dhaka, Bangladesh. Authentication of *Euphorbia hirta* was done from *Bangladesh National Herbarium* and the voucher specimen (authentication number: 43,100) was deposited in the herbarium for future references. Collected plants were washed properly and then the leaves were separated from the plant and other contamination, separated leaves ware then Shade dried. The dried plant parts were then grinded into coarse powder materials by milling from Pharmacognosy & Phytochemistry Lab of Primeasia University in order to be used for experiment.

### Drugs and chemicals

Povidone iodine ointment was obtained from the pharmaceutical industry Eskayef Bangladesh Limited. Alloxan monohydrate was obtained from Sigma Aldrich Chemicals, Germany. All other chemicals were obtained from Merck (Darmstadt, Germany) and were of analytical grade.

### Extraction procedure

Extraction was done by Macration, About 200 g of prepared coarse powder of leaves of *Euphorbia hirta* was soaked by 95% Ethanol (600 ml) in water in a conical flask and plugged with cotton and then covered with aluminium foil for seven days with constant stirring. After seven days the preparation was filtered and filtrate was collected for the purpose of preparing extract. The filtrate was evaporated by rotary evaporator at 78.37 °C and then the remaining part was kept in normal air for few days to evaporate any remaining solvent. The residue of *Euphorbia hirta* (39 g, 19.5%) was then collected, weighed and stored in a close container.

### Phytochemical analysis

Plants contain many chemical constituents which are therapeutically active or inactive like carbohydrates, triterpenoids, alkaloids, glycosides, tannins, flavonoids, essential oils and other similar secondary metabolites. Different qualitative chemical tests were performed for the present work to establish the profile of ethanolic extract for its chemical composition. Qualitative phytochemical analyses were done using the standard procedures [22, 23]. The total phenolic content (TPC) of Ethanolic Extract of *Euphorbia hirta* was determined using the Folin-Ciocalteu reagent [24] and total flavonoid content (TFC) was determined by the method of Rao et al. (2010) [25].

### Preparation of ointment

Hydrophobic ointments of povidone iodine and *Euphorbi hirta* was prepared by standard method [26]. Detail of ingredients for the formulation of ointment from *E. hirta* leaves is presented in Table 1.

**Table 1:** Preparation of medicated formulations with ethanolic extract of *E. hirta* leaves

Formula	Ingridients	Amount
5% *w*/w ointment Preparation	Povidone iodine **/** Ethanolic Extract of *Euphorbia hirta* whole plants.	5 g
	Petroleum jelly	70 g
	Cetostearyl alcohl	10 g
	PEG 6000	5 g
	Liquid paraffin	10 g
	Methyl paraben	1 Drop
	Total = 100 g	
10% *w*/w ointment Preparation	Povidone iodine **/** Ethanolic Extract of *Euphorbia hirta* leaves	10 g
	Petroleum jelly	65 g
	Cetostearyl alcohl	10 g
	PEG 6000	5 g
	Liquid paraffin	10 g
	Methyl paraben	1 Drop
	Total = 100 g	

### Pharmacological evaluation

#### Experimental animal

Female Swiss albino rats weighing between 180 to 200 g were used. The rats were collected from Pharmacy Department of Jahangirnagar University. They were housed in polypropylene cages in groups of six rats per cage and were kept in a room maintained at 25 ± 2 °C with a 12 h light-dark cycle, and were allowed to acclimatize for one week before the experiments. They were given free access to standard laboratory animal feed and water ad libitum. The rats were fasted over night before the experiment. All surgery was performed under isoflurane (5% in 100% oxygen) anesthesia, and all efforts were made to minimize suffering. Animal care and research protocols were based on principles and guidelines approved by the Guide for the Care and Use of Laboratory Animals (NIH publication No: 85–23, revised in 1985) [27]. The prior approval for conducting the experiments on rats was obtained from the Departmental Ethics Committee of Biomedical Research Center Dhaka University (Ref no. DPT/BMRC/2015–2016/313).

#### Acute skin irritation study of ointment formulation

The ethanolic extract of *E. hirta* was evaluated for anti-inflammatory activity that was performed on animals by using a standard protocol [21]. On doing this, one day prior to the experiment the animals were isolated in order to avoid contact with other rats and their dorsal hairs at the back were removed. One square cm area of whole and wounded skin of animals was subjected to different concentrations of 50 mg Ointment and they were observed for seven days to identify any signs of oedema and erythema. 0.8% formalin solution was used as standard irritant.

#### Induction of severe diabetes

The animals were injected by intraperitoneal route with a single dose of Alloxan monohydrate (120 mg/kg, body weight) in normal saline [28]. After 3 days Fasting blood glucose level was measured by Accu chek glucometer to confirm the diabetic status of the animals. The animals showing diabetes (Blood glucose level > 200 mg/dL) was selected for wound healing study using standard.

#### Excision wound model

All surgical affairs were conducted under sterile condition. The rats were anaesthetized prior to development of the wounds and hairs from the dorsal thoracic central region of anaesthetized rats were removed followed by marking the area of the wound at the back of the animals [29]. The wound was created along these markings by using toothed forceps, a surgical blade and pointed scissors. The thickness of the excision wound was 2.5 cm in width (circular area 4.90 cm^2^) and 0.2 cm depth which left open [30, 31]. The measurements of the wound areas of the excision wound model were taken on 1st, 4th, 8th, 12th and 16th day following the initial wound using transparent paper and a permanent marker. The recorded wound areas were measured by tracing the wound margin on graph paper. The wound contraction was calculated as percent reduction in wound area given in the formula below [32].

$$ {\displaystyle \begin{array}{l}\%\mathrm{Wound}\  \mathrm{contraction}=\frac{\mathrm{healed}\  \mathrm{area}}{\mathrm{Total}\  \mathrm{wound}\  \mathrm{area}}\times 100\\ {}\left(\mathrm{Healed}\  \mathrm{area}=\mathrm{original}\  \mathrm{wound}\  \mathrm{area}-\mathrm{present}\  \mathrm{wound}\  \mathrm{area}\right)\end{array}} $$

Epithelialisation time (day of fall of eschar and scar area) was noted as a number of days after wounding [7]. Significance in wound healing of the test groups has been derived by comparing healed wound area with that of the control group within the respective days. The experimental animals were divided into the following groups and received the subsequent treatments accordingly:

**Table:** 

Groups	Treatment
Group I	5 ml/kg/day distilled water, p.o.; (Normal control, NC)
Group II	Alloxan monohydrate (120 mg/kg, body weight) induced diabetes + Excision wound model (Diabetic wound control)
Group III	Alloxan monohydrate (120 mg/kg, body weight) induced diabetes + Excision wound model + 100 mg/kg/day Ethanolic extract of *E hirta*, p.o.
Group IV	Alloxan monohydrate (120 mg/kg, body weight) induced diabetes + Excision wound model + 200 mg/kg/day Ethanolic extract of *E. hirta*, p.o.
Group V	Alloxan monohydrate (120 mg/kg, body weight) induced diabetes + Excision wound model + 400 mg/kg/day Ethanolic extract of *E. hirta*, p.o.
Group VI	Alloxan monohydrate (120 mg/kg, body weight) induced diabetes + Excision wound model + 5% Povidone Iodine ointment
Group VII	Alloxan monohydrate (120 mg/kg, body weight) induced diabetes + Excision wound model + 5% Ointment of ethanolic extract of *E. hirta*
Group VIII	Alloxan monohydrate (120 mg/kg, body weight) induced diabetes + Excision wound model + 10% Ointment of ethanolic extract of *E. hirta.*

#### Collection of blood sample

After 16 days of the induction of diabetes, all animals of different groups were fasted overnight before collection of blood by retro orbital puncture into heparinized tubes under light and under anesthetic condition. The plasma was obtained by centrifugation at 5000 rpm for 5 min. The plasma was later on used for the determination of biochemical parameters like plasma nitric oxide and malondialdehyde level.

#### Evaluation of nitric oxide in plasma

As nitrite and nitrate are formed as end products of reactive nitrogen intermediates, the measurement of nitrite (free radicals) in rat plasma by using Griess method since the reagent acts as a marker to determine the formation of NO (an oxygen derived radical) [33]. 750 μl of each experimental sample was taken in a test tube in duplicate and 750 μl of standard sodium nitrite (50, 25, 12.5, 6.25, 3.125 and 1.56 μM/ml) was placed in different test tube, in duplicate. After this 750 μl of the sulphanilamide solution (1% Sulphanilamide in 5% phosphoric acid) was dispensed to all the test tube containing experimental samples and nitrite standards which were incubated for 5–10 min at room temperature. Following this 750 μl of the NED, 0.1% N-1-Napthylethylenediamine dihydrochloride in distilled water solution, was dispensed in all the test tube. The standard and samples were incubated again for 5–10 min at room temperature. A purple/magenta color appeared immediately. Absorbance was measured within 30 min in an ELISA plate reader with a filter of 520 nm and was expressed as μmol/ml. NO concentration was calculated by using standard curve equation.

#### Evaluation of malondialdehyde (MDA) in plasma

Malondialdehyde, a naturally occuring end product of membrane lipid peroxidation, is one of the most frequently used biomarker for free radical mediated damage [34, 35]. In the present study, plasma MDA level of rats was measured by TBARS (Thiobarbituric Acid Reacting Substances) method [36]. 1.5 ml of 0.67% thiobarbituric acid, 1.5 ml of 20% trichloroacetic acid and 700 μl of phosphate buffer, (pH 7.4) were added to 300 μl of serum and mixed followed by incubation in a water bath at 90 °C for 30 min. The reaction was stopped by dipping the test tubes into ice for 10 min. Samples were centrifuged at 3000 rpm. The supernatant was removed and absorbance was measured spectrophotometrically at 535 nm in a 1 cm cuvette. The concentration of MDA was calculated based on the absorbance.

#### Histopathology

For histopathological examination, the rats were sacrificed after 16 days before which blood was collected to obtain serum and excised immediately. Organs (heart, lung, liver, kidney, pancrease) and a specimen sample of tissue (5 μm thick) from the healed wound is isolated from each experimental group of rats and fixed in 10% buffer formalin for histopathological examination. After that histopathological evaluation of these organs and skin samples were done in Exim Bank Hospital, Department of histopathology, Dhaka, Bangladesh.

#### Statistical analysis

The values are represented as mean ± S.E.M, and statistical significance between treated and control groups was analyzed using one-way analysis of variance (ANOVA), followed by Dunnett’s test where *P* < 0.05 was considered statistically significant.

## Results

### Preliminary phytochemical analysis

The traditional use of the species was scientifically validated through the identification of the phytochemicals responsible for their use in indigenous systems of health care. The result of qualitative chemical analysis of the ethanolic extract of *E. hirta* is tabulated in Table 2.

**Table 2:** Preliminary phytochemical analysis of *E. hirta* leaves extract

Phytoconstituents	Ethanolic extract of *E. hirta*
Carbohydrates	+
Saponins	+
Alkaloids	+
Glycosides	-
Terpenoids	+
Steroids	+
Flavonoids	+
Phenolics and Tannins	+
Anthraquinone	-
Coumarins	+

+ = Present, - = Absent

### Quantitative evaluation for total polyphenolics and flavonoid contents

The plant is rich in phenolics, alkaloids, steroids, terpenoids and flavonoids as shown by preliminary phytochemical screening. The content of total phenols in dry extracts was estimated and expressed as gallic acid equivalents (GAE) per gram of dry extract. Total phenolic content of was 585 ± 2.18 mg GAE/g of dry extract. The standard curve equation of gallic acid was y = 0.003× + 0.1559 with a correlation coefficient of R^2^ = 0.9823. The content of total flavonoids in dry extracts was estimated and expressed as quercetin equivalents (QE) per gram of dry extracts. Total flavonoids content of EEH was 153.88 ± 2.15 mg QE/g of dry extract. The standard curve equation of quercetin was y = 0.0043× + 0.3437 with a correlation coefficient of R^2^ = 0.97.

### Acute skin irritation study

**Fig. 1:**
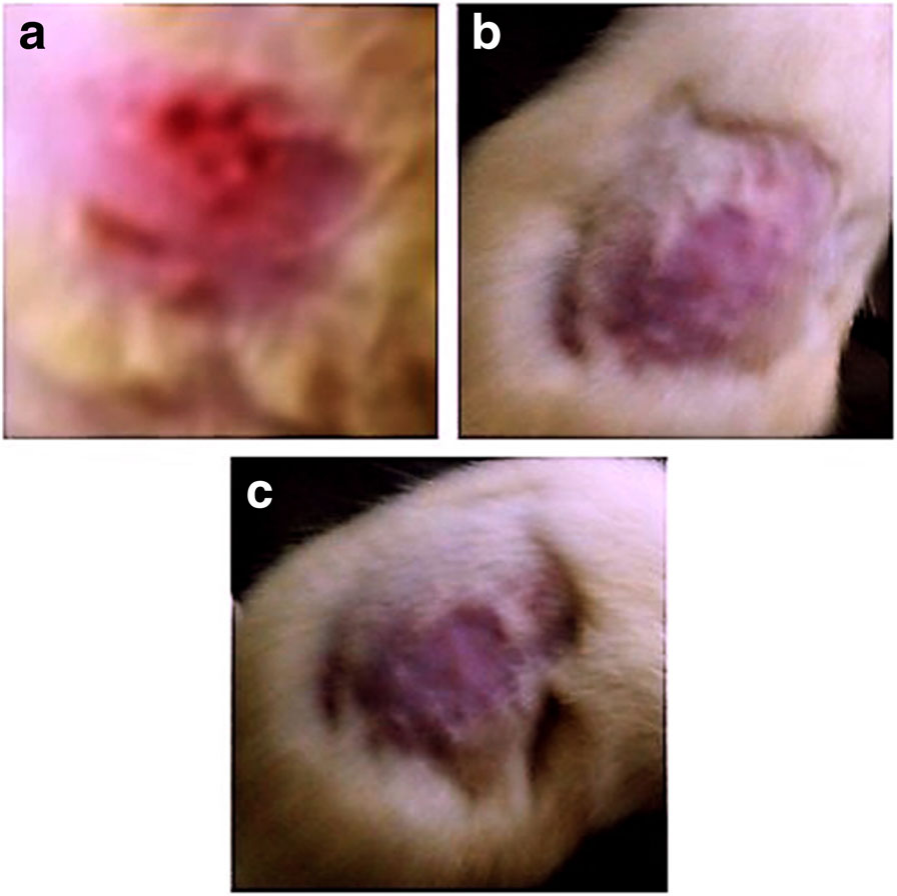
Photographic representation of effect of *E. hirta* ointment at the different concentrations over skin after 7 days where **a** = Group 1: Aqueous solution of 0.8% formalin, erythema and edema skin rash and inflammation was observed. **b** = Group 2: 50 mg of 5%*w*/w ethanolic extract of *E. hirta* whole plant ointment, no lesion occur. **c** = Group 3: 50 mg of 10% *w*/w ethanolic extract of *E. hirta* whole plant ointment, no lesion occur

All ointment formulations did not produce any skin irritation, i.e., erythema and edema for about a week when applied over the skin. However, 0.8% formalin group showed severe redness and edema as shown in Fig. 1.

### Wound healing effect of *E. hirta* on diabetic excision wound model

**Fig. 2:**
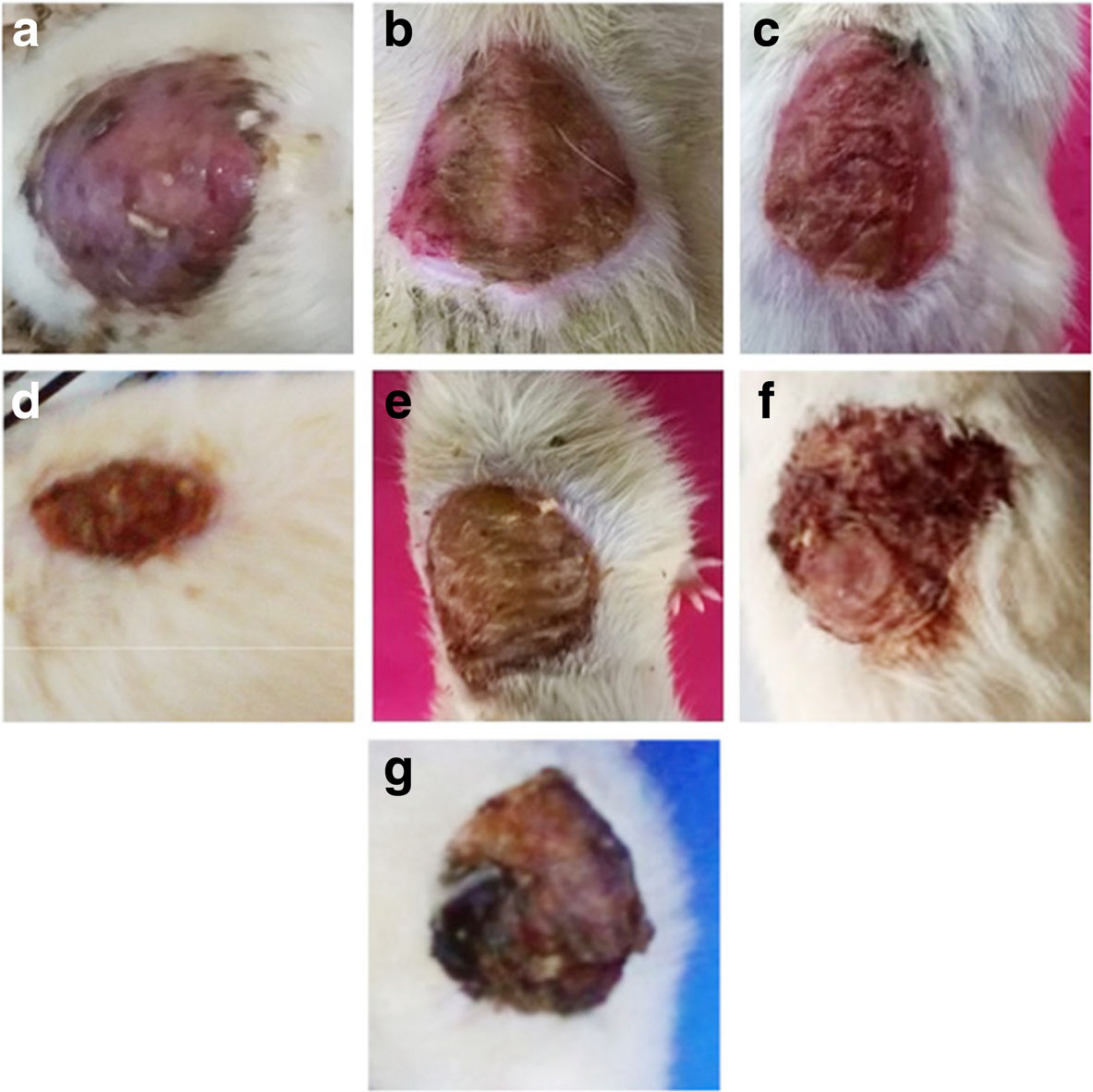
Photographic representation of effect of *E. hita* on the wound contraction at the end of the study showing wound healing after oral and topical treatment at the end of the study. **a**: Group II = Diabetic wound control, **b**: Group III = Diabetic wound control +100 mg ethanolic extract of *E. hirta*, **c**: Group IV = Diabetic wound control +200 mg ethanolic extract of *E. hirta*, **d**: Group V = Diabetic wound control +400 mg ethanolic extract of *E. hirta*, **e**: Group VI = Diabetic wound control +5% Povidone iodine, **f**: Group VII = Diabetic wound control +5% ethanolic extract of *E. hirta*, **g**: Group VIII = Diabetic wound control +10% ethanolic extract of *E. hirta*

Oral administration (100, 200 and 400 mg/kg, p.o.) and topical application (5% and 10% ointment) of *E. hirta* whole plant extract promotes the contraction of wound in diabetic rats, when compared to diabetic control (Fig. 2). The percentage of wound contraction on 16 day of observation showed that in diabetic control it was 6.12%. In the orally treated groups it was found to be 35.92, 44.69 and 61.42% at different doses (100, 200 and 400 mg/kg). Moreover, topical application of 5 and 10% of *E. hirta* whole plant extract the percentage of wound contraction was 32.86 and 36.32%, respectively whereas in standard drug, povidone iodine ointment, it was 39.79%. Observation showed that on 8th day onwards, the oral administration and topical applications of ethanolic extract of *E. hirta* promoted the wound contraction faster than diabetic control. Wound contraction progressed much faster in orally treated groups than the topically treated group and diabetic control group. The results of wound healing activity and % (percentage) of wound contraction of *E. hirta* whole plant extract in alloxan induced diabetic rats are shown in Table 3.

**Table 3:** Effect of ethanolic extract of *E. hirta* whole plant on excision wound in alloxan induced diabetic rats

Group/ Post wounding days	Wound area (cm^2^)					% of wound contraction at the end of the study
	1st day	4th day	8th day	12th day	16th day	
Group I	N/A	N/A	N/A	N/A	N/A	N/A
Group II	4.90 ± 1.13	4. 90 ± 1.20	4.85 ± 0.88	4.72 ± 0.89	4.60 ± 0.93	6.12
Group III	4.90 ± 1.11	4.83 ± 1.05^†^	4.58 ± 1.17^†^	3.76 ± 0.59^†^	3.14 ± 0.36^†^	35.92
Group IV	4.90 ± 1.03	4.70 ± 0.91^†^	4.50 ± 0.86^†^	3.23 ± 0.65^†^	2.71 ± 0.40^*^	44.69
Group V	4.90 ± 1.10	4.58 ± 1.07^†^	4.16 ± 0.88^†^	2.48 ± 0.33^*^	1.89 ± 0.35^**^	61.42
Group VI	4.90 ± 1.12	4.51 ± 1.01^†^	4.35 ± 0.76^†^	3.26 ± 0.33^†^	2.95 ± 0.54^*^	39.79
Group VII	4.90 ± 1.16	4.83 ± 1.21^†^	4.55 ± 0.73^†^	3.92 ± 0.58^†^	3.29 ± 0.37^†^	32.86
Group VIII	4.90 ± 0.88	4.75 ± 1.03^†^	4.47 ± 0.68^†^	3.41 ± 0.56^†^	3.12 ± 0.49^†^	36.32

Each value is Mean ± S.E.M (*n =* 3). (*) indicates statistically significant difference from the diabetic wound control using ANOVA, followed by Dunnett’s multiple comparison test (^**^ *p* < 0.01). (†) indicates statistically no significant difference from the diabetic wound control using ANOVA, followed by Dunnett’s multiple comparison test (*p* > 0.05). Group I = normal control, Group II = Diabetic wound control, Group III = Diabetic wound control +100 mg ethanolic extract of *E. hirta*, Group IV = Diabetic wound control +200 mg ethanolic extract of *E. hirta*, Group V = Diabetic wound control +400 mg ethanolic extract of *E. hirta*, Group VI = Diabetic wound control +5% Povidone iodine, Group VII = Diabetic wound control +5% ethanolic extract of *E. hirta*, Group VIII = Diabetic wound control +10% ethanolic extract of *E. hirta*

The statistical analysis showed that reference drugs and extract (200 and 400 mg/kg, p.o) caused a significant contraction of the wound as compared to diabetic wound control group ((*p* < 0.5 ˗ *p* < 0.01)) at the end of the study period. There was significant increase in wound contraction observed with the oral administration of *E. hirta* whole plant extract at the doses of 200 and 400 mg/kg, p.o. in contrast with diabetic control group (*p* < 0.05 and *p* < 0.01, respectively). However, oral administration of *E. hirta* whole plant extract at the higher dose promoted wound contraction better than povidone iodine treated group but no significant difference was observed.

### Histopathological evaluation

#### Effect of EHEE on histopathology of skin, pancreas, kidney, liver and heart

##### Skin

Histology of excision biopsy of skin wound at day 16 showed healed skin structures with normal epithelization, restoration of adnexa and fibrosis within the dermis in the treated groups while the diabetic control group lags behind treated group in formation of the amount of ground substance in the granulation tissue, as observed in tissue sections.

**Fig. 3:**
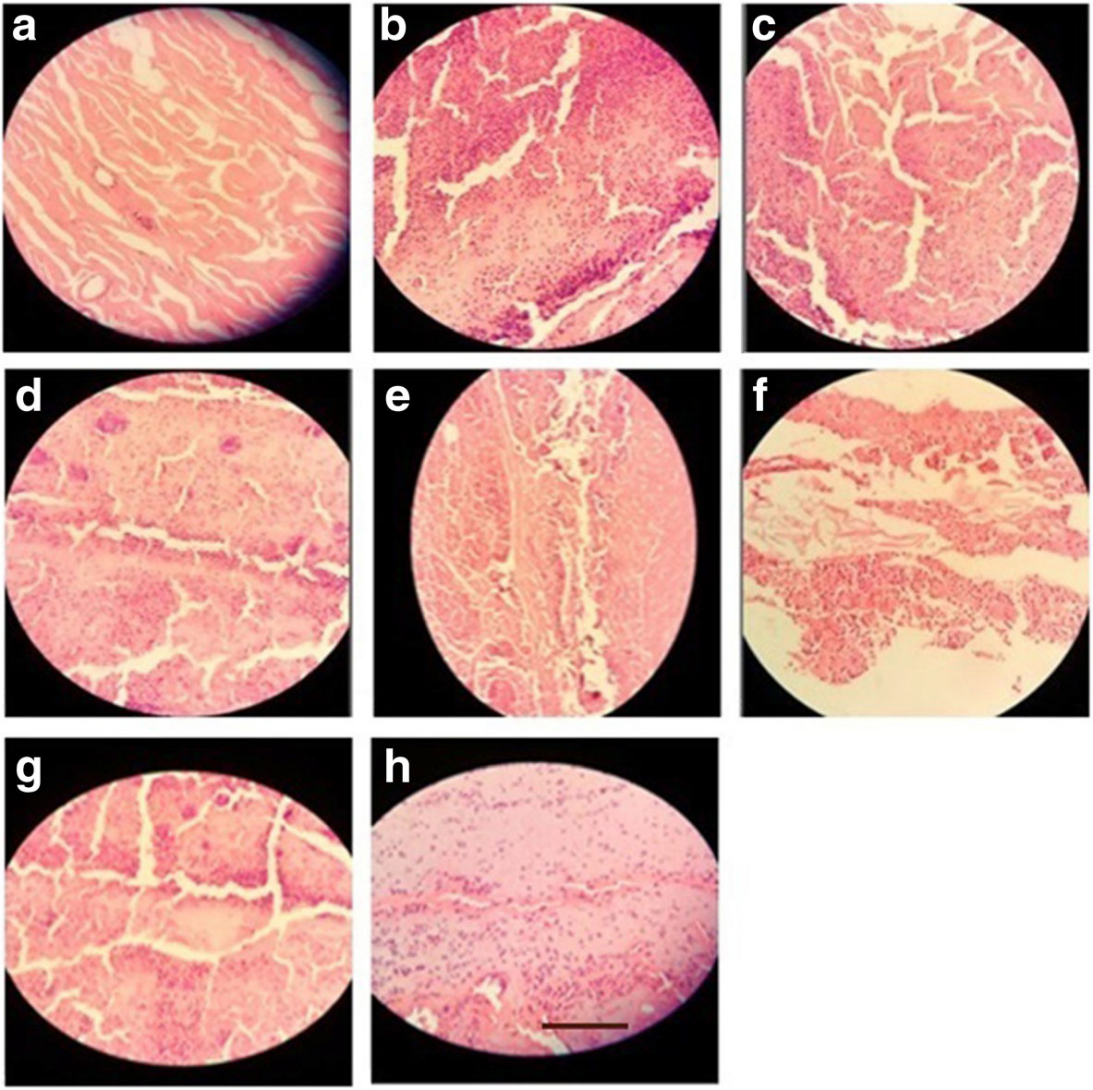
Histopathological changes in the skin; **a**: Group I = Normal control, **b**: Group II = Diabetic wound control, **c:** Group III = Diabetic wound control +100 mg ethanolic extract of *E. hirta*, **d**: Group IV = Diabetic wound control +200 mg ethanolic extract of *E. hirta*, **e**: Group V = Diabetic wound control +400 mg ethanolic extract of *E. hirta*, **f**: Group VI = Diabetic wound control +5% Povidone iodine, **g**: Group VII = Diabetic wound control +5% ethanolic extract of *E. hirta*, **h**: Group VIII = Diabetic wound control +10% ethanolic extract of *E. hirta*. Each group was assessed at 400X magnification, scale bar: 40 μm

*E. hirta* at the dose of 100 mg/kg, p.o. showed minimal amount of granulation tissue, large number of mononuclear inflammatory cells, and delayed epithelization whereas *E. hirta* at the dose of 200 mg/kg showing moderate amount of granulation tissue, few number of mononuclear inflammatory cells, and early epithelization. *E. hirta* at the dose of 400 mg/kg showing healed skin structures with well-formed, near to normal epidermis, restoration of extensive fibrosis and collagen tissue within the dermis. However, orally treated groups showed minimal amount of granulation tissue, mild to moderate number of mononuclear inflammatory cells infiltration as compared to the topically treated groups (Fig. 3a-f).

##### Pancreas

**Fig. 4:**
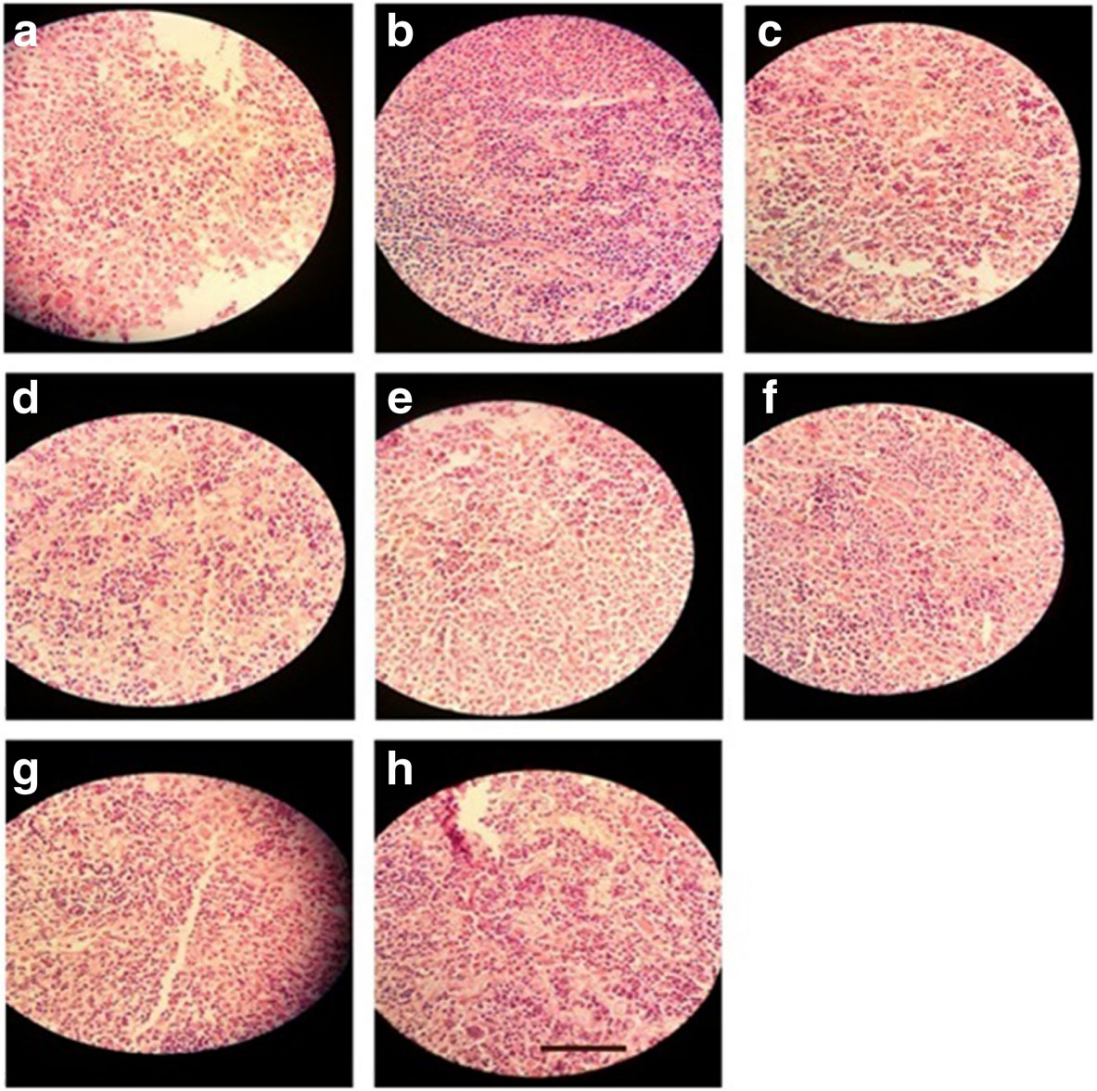
Histopathological changes in the pancrease; **a**: Group I = Normal control, **b**: Group II = Diabetic wound control, **c**: Group III = Diabetic wound control +100 mg ethanolic extract of *E. hirta*, **d**: Group IV = Diabetic wound control +200 mg ethanolic extract of *E. hirta*, **e**: Group V = Diabetic wound control +400 mg ethanolic extract of *E. hirta*, **f**: Group VI = Diabetic wound control +5% Povidone iodine, **g**: Group VII = Diabetic wound control +5% ethanolic extract of *E. hirta* **, h**: Group VIII = Diabetic wound control +10% ethanolic extract of *E. hirta.* Each group was assessed at 400X magnification, scale bar: 40 μm

Normal control rat exhibited normal histological architecture. Group received alloxane without treatment, demonstrated cellular damage to the pancreatic acini and islets, which showed pancreatic β-cell damage and degeneration. Diabetic rats treated with the EHEE at the doses of 100 and 200 mg/kg exhibited degenerative and atrophic changes characterized by β-cell vacuolation and marked to moderate reduction in their size, respectively. The groups treated with EHEE at the dose of 400 mg/kg, p.o. did not show any significant change of pancreas, when compared with normal pancreas. However, ointment treated groups did not significantly reduced cellular damage and showed mild to moderate multifocal mononuclear cell infiltration in the pancreas (Fig. 4a-f).

##### Liver

**Fig. 5:**
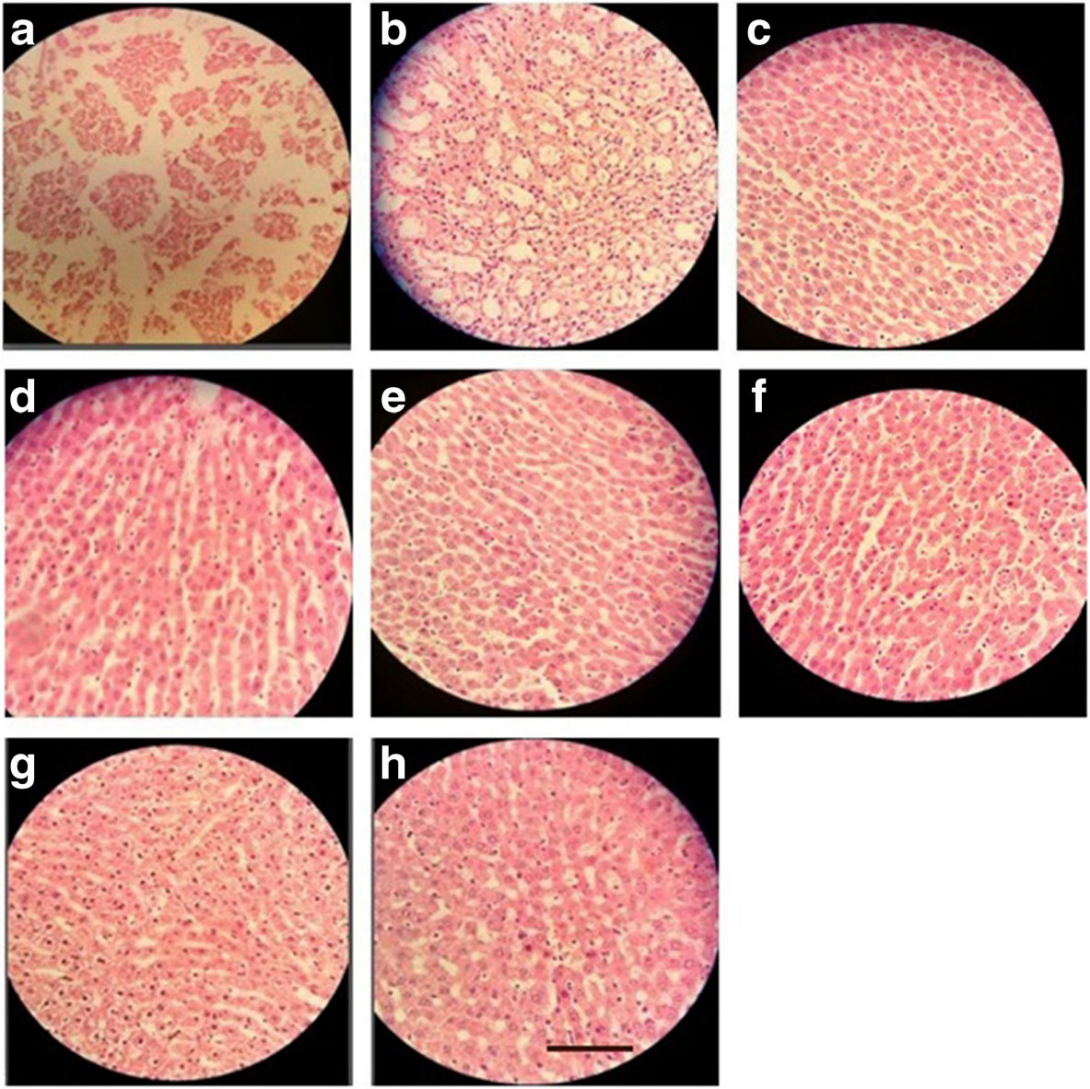
Histopathological changes in the liver; **a**: Group I = Normal control, **b**: Group II = Diabetic wound control, **c**: Group III = Diabetic wound control +100 mg ethanolic extract of *E. hirta*, **d**: Group IV = Diabetic wound control +200 mg ethanolic extract of *E. hirta*, **e**: Group V = Diabetic wound control +400 mg ethanolic extract of *E. hirta*, **f**: Group VI = Diabetic wound control +5% Povidone iodine, **g**: Group VII = Diabetic wound control +5% ethanolic extract of *E. hirta*, **h**: Group VIII = Diabetic wound control +10% ethanolic extract of *E. hirta.* Each group was assessed at 400X magnification, scale bar: 40 μm

Liver tissue of diabetic rat showed distortion in the arrangement of cells around the central vein, enlargement and thickening of the walls of veins, capillaries, and development of fibrosis in the degenerated cells. EHEE (400 mg/kg, p.o.) treatment almost restored the cellular arrangement of hepatocytes around the central vein and reduced fibrosis. It also helped to bring the blood vessels to normal condition. On the other hand, ointment treated groups did not effectively restored the cellular arrangement of hepatocytes (Fig. 5a-f).

##### Kidney

**Fig. 6:**
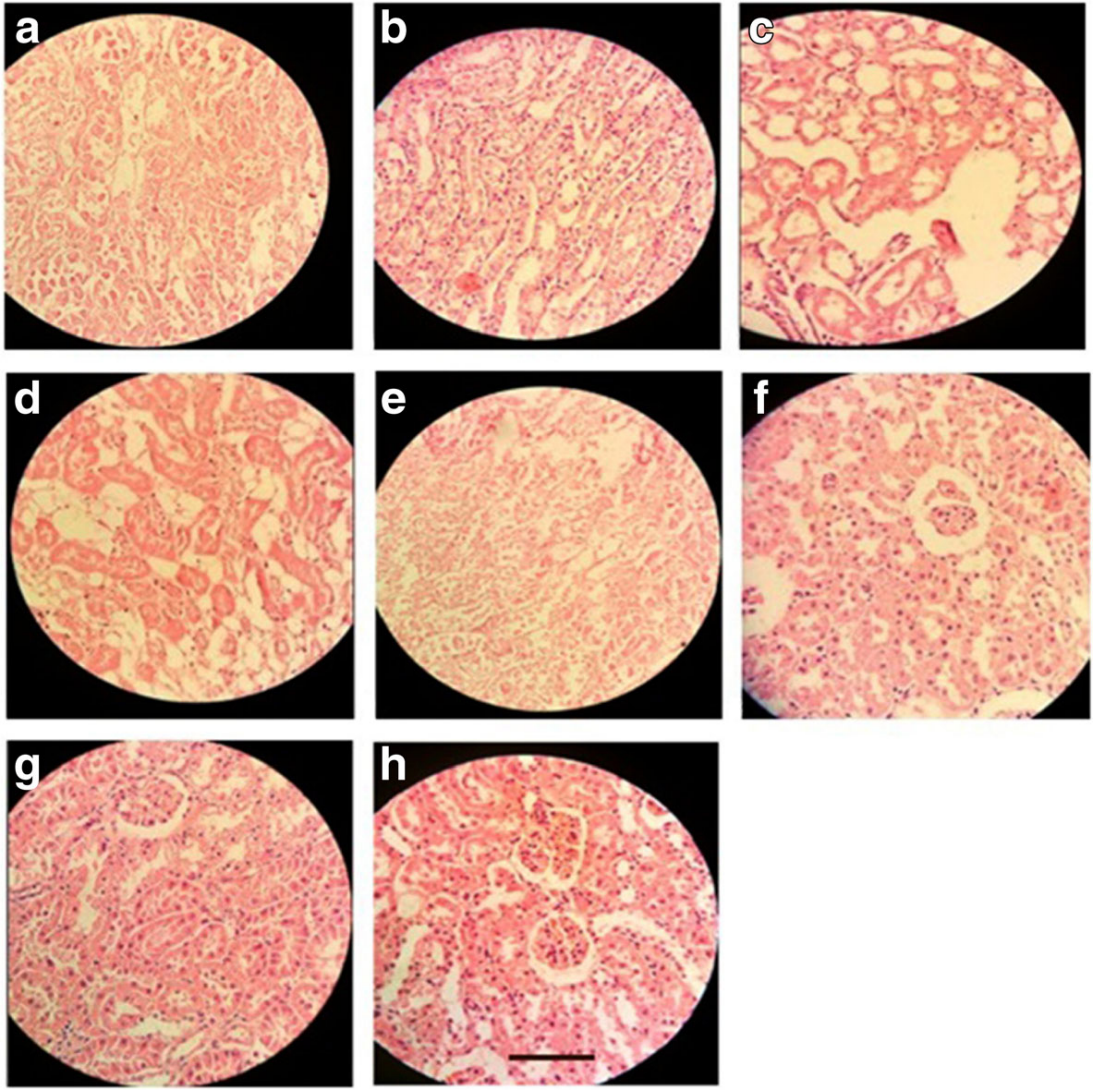
Histopathological changes in the right kidney; **a**: Group I = Normal control, **b**: Group II = Diabetic wound control, **c**: Group III = Diabetic wound control +100 mg ethanolic extract of *E. hirta*, **d**: Group IV = Diabetic wound control +200 mg ethanolic extract of *E. hirta*, **e**: Group V = Diabetic wound control +400 mg ethanolic extract of *E. hirta*, **f**: Group VI = Diabetic wound control +5% Povidone iodine, **g**: Group VII = Diabetic wound control +5% ethanolic extract of *E. hirta*, **h**: Group VIII = Diabetic wound control +10% ethanolic extract of *E. hirta*. Each group was assessed at 400X magnification, scale bar: 40 μm

Morphological features of kidney remains normal in the control group like prominent glomeruli, collecting ducts, tubules and ascending and descending loops. Aloxane-induced DM group showed presence of crystal deposition along with destructed glomeruli and infiltration of red blood cells. Groups received the EHEE orally demonstrated the reversal of these pathological destructions as apparent by the cell regeneration and moderate to minimal infiltration of inflammatory cells. However, ointment treated groups did not significantly reduced the pathological destructions showing inflammatory cells infiltration (Fig. 6a-f).

##### Heart

**Fig. 7:**
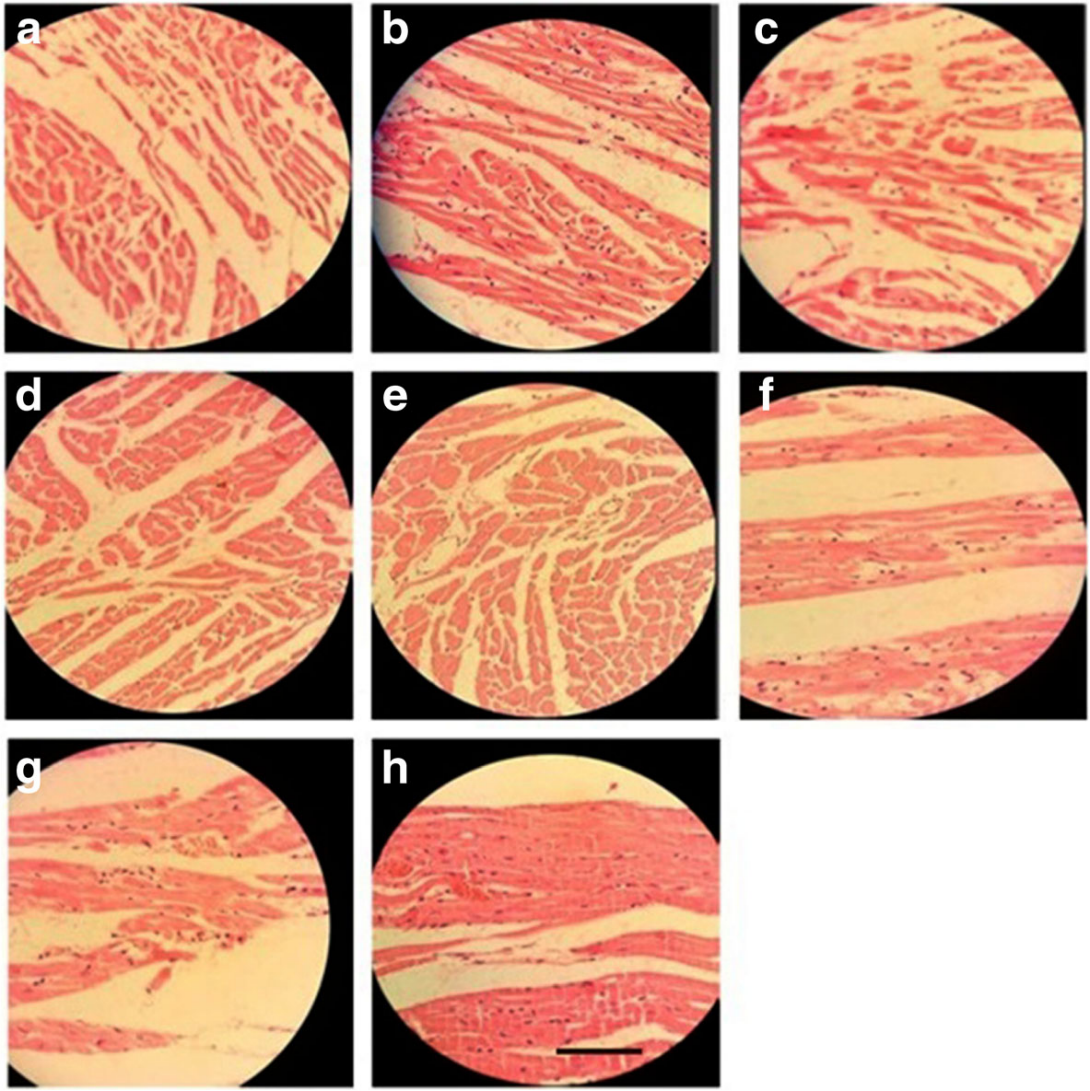
Histopathological changes in the heart; **a**: Group I = Normal control, **b**: Group II = Diabetic wound control, **c**: Group III = Diabetic wound control +100 mg ethanolic extract of *E. hirta*, **d**: Group IV = Diabetic wound control +200 mg ethanolic extract of *E. hirta*, **e**: Group V = Diabetic wound control +400 mg ethanolic extract of *E. hirta*, **f**: Group VI = Diabetic wound control +5% Povidone iodine, **g**: Group VII = Diabetic wound control +5% ethanolic extract of *E. hirta*, **h**: Group VIII = Diabetic wound control +10% ethanolic extract of *E. hirta*. Each group was assessed at 400X magnification, scale bar: 40 μm

Normal control group showed a regular arrangement of cardiac myocytes. Alloxane-induced DM rats demonstrated a large infarct area with prominent lymphocyte infiltration and fibrosis. Oral administration of EHEE reversed these morphological changes in a dose dependent manner and better result was observed in the orally treated group as compared to the ointment treated groups (Fig. 7a-f).

#### Effect of ethanolic extract of *E. hirta* on biochemical parameters

##### Fasting blood glucose level

The administration of single dose of alloxan produced significant increase in the blood glucose levels in all the groups as compared to the normal control group. However, oral administration of *E*. *hirta* ethanolic extract significantly reduced the blood glucose levels in diabetic wound rats (*p* < 0.01) on day 8 and day 16 as compared to the diabetic wound control (*p* < 0.01). On the other hand, topical application of *E. hirta* did not influence the blood glucose levels in diabetic rats (*p* > 0.05) (Table 4).

**Table 4:** Effect of ethanolic extracts of *E. hirta* on the fasting plasma blood glucose level of experimental rats

Groups/ Post wounding days	Blood glucose level(mg/dl)		
	1st day	8th day	16th day
Group I	85.70 ± 2.18	83.05 ± 1.98	86.40 ± 2.16
Group II	225.00 ± 3.02^a**^	270.00 ± 3.22^a**^	284.79 ± 5.83 ^a**^
Group III	227.50 ± 2.77^a**^	195.50 ± 2.40^b**^	163.00 ± 3.10 ^b**^
Group IV	239.40 ± 3.19^a**^	176.70 ± 2.65 ^b**^	159.40 ± 3.74 ^b**^
Group V	240.20 ± 2.45^a**^	147.60 ± 2.98 ^b**^	109.00 ± 2.87 ^b**^
Group VI	234.00 ± 1.64^a**^	250.10 ± 2.75^b†^	277.00 ± 4.22 ^b†^
Group VII	219.20 ± 2.41^a**^	245.30 ± 2.25 ^b†^	266.40 ± 3.37 ^b†^
Group VIII	210.40 ± 2.15^a**^	246.70 ± 2.90 ^b†^	269.80 ± 5.18 ^b†^

Each value is Mean ± S.E.M (*n =* 3). (*) indicates statistically significant difference from respective group using ANOVA, followed by Dunnett’s multiple comparison test (^**^ *p* < 0.01). (†) indicates statistically no significant difference from respective group using ANOVA, followed by Dunnett’s multiple comparison test (*p* > 0.05). a = when compared with Normal control, b = when compared with diabetic wound control. Group I = normal control, Group II = Diabetic wound control, Group III = Diabetic wound control +100 mg ethanolic extract of *E. hirta*, Group IV = Diabetic wound control +200 mg ethanolic extract of *E. hirta*, Group V = Diabetic wound control +400 mg ethanolic extract of *E. hirta*, Group VI = Diabetic wound control +5% Povidone iodine, Group VII = Diabetic wound control +5% ethanolic extract of *E. hirta*, Group VIII = Diabetic wound control +10% ethanolic extract of *E. hirta*

##### Estimation of free radicals (MDA and NO levels)

**Fig. 8:**
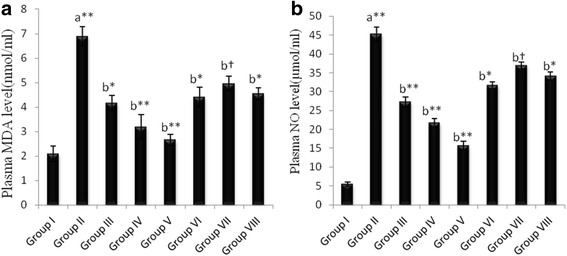
Effect of *E. hirta* on plasma malondialdehyde (**a**) and nitric oxide (**b**) levels. Each value is Mean ± S.E.M (*n =* 3). (*) indicates statistically significant difference from respective group using ANOVA, followed by Dunnett’s multiple comparison test (^**^ *p* < 0.01). (†) indicates statistically no significant difference from respective group using ANOVA, followed by Dunnett’s multiple comparison test (*p* > 0.05). a = when compared with Normal control, b = when compared with diabetic wound control. Group I = normal control, Group II = Diabetic wound control, Group III = Diabetic wound control +100 mg ethanolic extract of *E. hirta*, Group IV = Diabetic wound control +200 mg ethanolic extract of *E. hirta*, Group V = Diabetic wound control +400 mg ethanolic extract of *E. hirta*, Group VI = Diabetic wound control +5% Povidone iodine, Group VII = Diabetic wound control +5% ethanolic extract of *E. hirta*, Group VIII = Diabetic wound control +10% ethanolic extract of *E. hirta.*

In contrast with the normal control group, diabetic wound significantly increased free radicals (LPO and NO level) levels in diabetic wound control group. However, treatment with ethanolic extract of *E. hirta* showed significant decrease in the level of free radicals, LPO and NO as compared with diabetic wound control. Moreover, in oral administration of ethanolic extract of *E. hirta* showed significant decrease in all the groups (*p* < 0.01). On the other hand, compared to the ointment formulation orally administered ethanolic extract of *E. hirta* resulted in better inhibition on the elevated levels of MDA and NO has been shown in Fig. 8.

## Discussion

On phytochemical screening, EHEE showed the presence of alkaloids, phenols, steroids, triterpinoids and flavonoids. A number of scientific studies have reported that certain terpenoids, steroids and phenolic compounds (tannins, coumarins and flavonoids) have protective effects due to their antioxidant properties [37] These active constituents support the process of wound healing by increasing the viability of collagen fibrils, by enhancing the strength of collagen fibres either by augmenting the circulation or by preventing the cell damage or by promoting DNA synthesis [38]. The major constituents like flavonoids, triterpenoids and alkaloids present in EEH might play a major role in the process of wound healing, however further phytochemical studies are needed to find out the active compound(s) responsible for wound healing promoting activity.

Wound healing is associated with a series of physiological processes that include inflammation, cell proliferation and migration, angiogenesis, matrix synthesis, re-epithelialization and formation of granulation tissues [39]. The new tissues are composed of fibroblasts, collagen, edema and new small blood vessels [40]. A safe treatment for wound healing should ideally consider a therapeutic agent that will improve new tissue formation without producing any deleterious side effect. Plant products are universally accepted as potential wound healers for years due to their profound availability with minimal toxicity, lack of undesirable side effects and their potentiality as crud preparation [39]. In our present study involving excision wound models in diabetic condition which included observation of different physical, histological and biochemical parameters, indicated the wound healing activity in the ethanolic extract of *E. hirta*. The study revealed significant increase in blood glucose level was observed in alloxan induced diabetic rats. Alloxan cause immense reduction in insulin release by the destruction of beta cells of the islets of langerhans and caused hyperglycemia. The adipose tissue and skeletal muscle are unable to uptake glucose from serum in the absence of insulin so glucose conversion to fat and glycogen is blocked in the adipose tissue and skeletal muscles, resulting in increase of blood glucose level [41]. In the current study, the healing of skin wounds was delayed significantly in diabetic rats, which may be correlated with the elevated glucose levels measured in the blood plasma of these animals. As expected, the diabetic conditions of the rats favored in delaying the wound healing process by abnormal physiological response. Significant decrease in the body weight gained by the diabetic animals indicated impaired metabolic activities and may also be due to irregular absorption and elimination of wastes. The hyperglycemic status triggered the animals to become catabolic by disintegrating protein and fat due to minimum accessibility of glucose for cell nutrition, which had not supported the actual healing process [42]. It was observed that the oral administration of *E. hirta* decreased the blood glucose levels significantly after 16 days. The present work demonstrated that the *E. hirta* ethanolic extract accelerate wound healing in diabetes. The results suggested that treatment with *E. hirta* ethanolic extract may be beneficial for different stages of wound healing process, i.e. synthesis of fibrous tissues along with synthesis and contraction of collagen, resulting in faster healing and also exhibits hypoglycemic activity since a control over blood glucose levels has been shown to improve wound healing in diabetics.

A significant reduction in fasting blood glucose has been observed in oral administration of *E. hirta* ethanolic extract at a dose of 400 mg/kg body weight per day to alloxan-induced diabetic rats for a period of 16 days. On the other hand, topical application of *E. hirta* at a dose 50 mg of 10% *w*/w per day to alloxane-induced diabetic rats for a period of 16 days resulted in no significant change in fasting blood glucose levels. Therefore, the highest percent of wound closer was observe in the group treated orally the ethanolic extract of *E. hirta* at the dose of 400 mg/kg body weight per day*.*

Experimental and clinical evidences suggest that chronic wound undergoes substantial oxidative stress by neutrophils derived oxidants and free radicals, both of which contribute markedly to tissue damage during chronic wound inflammation [26]. Excessive formation of reactive oxygen species (ROS) leads to oxidative stress followed by cytotoxicity and delayed wound healing. As a result, eradication of ROS could be a significant approach for the healing of chronic wound [29]. Therefore, estimation of free radicals like MDA and NO in plasma is relevant because the free radicals have been reported to delay wound healing. Our study revealed that the ethanolic extract of *E. hirta* had significant reducing effect on free radicals stress and helped to prevent inflammation and oxidative damage which ultimately promote wound healing process.

The results of biochemical parameters were further supported by the histopathological changes of different organs (liver, pancrease, kidney, heart and skin from wound area). The histopathological examination reported the involvement of oxidative stress which implicated in the progression of complications of DM (diabetes mellitus), this may also lead to second complications such as hepatotoxicity, nephropathy, neuropathy and coronary heart disease [43].

## Conclusion

In conclusion, the present study revealed that ethanolic extract of E*. hirta whole plant* could improve wound healing after topical and oral administration. Moreover, orally treated groups showed better diabetic wound healing activity due to its hypoglycemic effect. The healing effects seemed to be due to decreased blood glucose and free radical generated tissue damage as the study reported that the oral administration of *E. hirta* ethanolic extract significantly reduces blood glucose level and plasma free radicals. It also significantly improves the wound ulcer healing on day 16. However, the ethanolic extract of *E. hirta* at the dose of 400 mg/kg of body weight was beneficial in alleviating histological injuries in diabetic wound animal tissues and organs. Thus, the results of the present study provide a scientific rationale for the use of *E.hirta* ethanolic extract, and show that the etanolic extract attenuated the diabetic wound in rats.

## Abbreviations

DM: Diabetes mellitus
*E*: *Euphorbia*
MDA: Malondialdehyde
NO: Nitric oxide
ROS: Reactive oxygen species
TFC: Total flavonoid content
TPC: The total phenolic content
WHO: World Health Organization
